# The Town Dweller

**Published:** 1890-06

**Authors:** 


*The Town Dweller.* By J. MILNER FOTHERGILL, M.D. London: H. K. LEWIS. 1889.

Dr. B. W. Richardson, who writes the introduction to this posthumous work by Dr. Fothergill, considers that it is an outline of a larger work contemplated by that voluminous writer. However that may be, it is, so far as it goes, complete in itself, and is written in Dr. Fothergill's vigorous and epigrammatic style so familiar to the medical reader.

Those who are acquainted with the wan appearance of many workers in town, condemned to sedentary occupations extending over long hours, will agree with the author about the need of an investigation into the causes which are bringing about the decadence of the town dweller, and will welcome the suggestiveness of this book towards providing the remedies. It deals with the subject under the headings of

"The Town Immigrant;" "His Dwelling;" "His Surroundings;" "The Air he Breathes;" "The Water he Drinks;" "The Food he Eats;" "His Beverages;" "His Work;" "His Amusements;" "His Mind and Body;" "His Progeny."

Evidenced by recent Acts of Parliament, which yet require much simplification, there are indications of an increasing public desire to grapple with the difficulties of the question; and the establishment of committees for the consideration of the better housing of the poor, and the recognition of the healthiness of permanent open spaces, are among the more hopeful of these.

Dr. Fothergill's small book has much that is useful for both rich and poor, and we hope our readers will study it for themselves.

